# A budding science of illegal markets

**DOI:** 10.1073/pnas.2511780123

**Published:** 2026-08-10

**Authors:** Jonathan P. Caulkins

**Affiliations:** ^a^ Heinz College, Carnegie Mellon University, Pittsburgh, PA 15237

**Keywords:** illegal markets, crime, trafficking, drug policy

## Abstract

Illegal markets create untold societal and environmental harm, but they have attracted surprisingly little scholarly attention. This paper explores illegal markets as a class of objects. It considers similarities and differences across markets, with particular attention to nine: human smuggling, commercial sex, firearms, money laundering, illegal fishing, international wildlife trafficking, counterfeit goods, drugs, and tobacco products. These nine markets display enormous variation, stemming in part from differences in the “physics” of the good or service they provide. Other differences can be seen as adaptations to different policy and enforcement pressures. Some markets have changed in fundamental ways over recent decades, while others remain more stable. The overall rate of true innovation is not necessarily high, but most appear quick to adopt innovations that are created by the larger economy such as cryptocurrencies, secure communications, and dark web distribution. Low rates of investment in research and development, branding, and marketing along with simultaneous high rates of adaptability may be a natural outgrowth of these markets being dominated by relatively small organizations. It is hypothesized that one can group the common illegal markets into those that: 1) Supply goods stolen from a common heritage, either natural (illegal fishing, wildlife trafficking, illegal mining) or cultural (antiquities); 2) Supply goods or services to people committing crimes beyond just consuming the banned products (guns, money laundering); 3) Undermine an authority’s rights (smuggling humans across borders, counterfeiting goods, tax-evading cigarettes); and 4) Supply products that harm the user and/or seller (drugs, commercial sex, gambling, raw milk).

## Significance

Illegal markets present important challenges to society and have distinctive features, but scientific research on them has mostly been piecemeal, one market at a time. This paper explores differences and commonalities across various illegal markets to understand them at a more fundamental level. It turns out that many salient characteristics of markets stem from the characteristics of that good, service, or policy environment, and are not inevitable consequences of illegality per se. However, all markets investigated here do share certain traits, including many that flow from the relative atomization of the organizations participating in these markets, and their noncorporate form.

This paper describes differences and commonalities across a number of important illegal markets and the supply chains that feed them. The method might be likened to a narrative review, but via direct interaction with experts, not literature synthesis, because so little has been written comparing across different illegal markets.

The raw material for these comparisons mostly comes from the February 2025 Policy and Research on Illegal Markets (PRIM) workshop held at RAND in Santa Monica. The workshop convened 17 experts on nine specific markets, or types of markets. They wrote the market-specific papers contained within this volume. In addition to direct interaction (reading and discussing paper drafts, etc.), the experts’ wisdom was also tapped by having them fill out a matrix describing for each market a total of 53 attributes grouped into seven categories (basics, customers and retail selling, supply chains, motivation for illegality, changes over time, enforcement against the market, and the state of research on the market). This paper incorporates statements about almost all of those attributes that are not more suitable to the companion paper by Paoli and Reuter which focuses on policy implications (1).

The nine markets are a purposive not a random sample, skewed toward those that are relatively large, important, and/or well-studied. The workshop participants have varying degrees of familiarity with other illegal markets (gambling, sand, human organs, raw milk, etc.), so reference is occasionally made to those. Statements that hold for all of the markets considered here should formally be construed only as hypotheses regarding the entire set of illegal markets, but the markets considered here may account for a sizable share, perhaps even the majority of, societal problems generated by all illegal markets combined.

The motivation here is scientific inquiry, not proposing or evaluating potential solutions to those market-driven problems. More specifically, the goal is to see what hypotheses about illegal markets overall emerge from applying inductive reasoning to rich descriptions of a number of specific illegal markets. However, policy-adjacent observations will be made where possible. For example, the following contrast between markets for drugs and wildlife products is intrinsically interesting, and happens also to be policy relevant. The core harms motivating prohibition for drugs mostly pertain to consumption (addiction, overdose, etc.), whereas for wildlife—including illegal fishing—the harms are mostly associated with production or extraction (environmental damage, decimation of populations, extinction risk, etc.). That matters when seizures are partially offset by increased production, and so lead to lower consumption but greater production (because production = consumption + seizures). Seizing cocaine may reduce consumption-related harm, but seizing ivory, rosewood, and pangolin scales may increase production and, hence, the core harms from wildlife trafficking.

There are many fascinating contrasts between different illegal markets, and recognizing differences can be instructive. For example, if a drug market expert argued that prohibitions necessarily create violent markets and overflowing prisons, because that often happens with drug prohibitions, it is useful to note that many other illegal markets spawn neither much violence nor mass incarceration.

There are not many universal traits shared by all of these illegal markets. Still the quest to identify shared traits is fruitful, even if challenging. Indeed, the pitch used to recruit the stellar set of experts who wrote the market-specific papers appearing in this volume included the following observation. Experts on any individual illegal market mostly interact with scholars interested in problems related to the product that market supplies. E.g., experts on illegal markets for tobacco products mostly interact with public health researchers who are interested in the epidemiology of smoking, or medical researchers interested in cancer. They tend not to interact with experts on other illegal markets. That would be comparable to experts on African elephants talking mostly to experts on the Serengeti and Tanzanian tourism, and experts on squirrels talking to experts on attic infestation and squirrel-proof bird feeder design. Siloing of expertise risks overgeneralization. Imagine elephant experts saying “I study elephants, so I understand mammals as a class. I know that mammals are all large animals with tusks” and squirrel experts saying “Based on my study of squirrels, I know that all mammals eat nuts and have bushy tails.”

Fortunately, taxonomists have developed a more fundamental understanding of mammals. Even though mammals come in an amazing variety of shapes and sizes, what distinguishes them from other animals is hair, milk production, and certain aspects of bone structure (e.g., the “hammer, anvil, and stirrup” ear bones).

One goal of this paper is to ask what analogs of hair, milk production, and ear bones do all or most illegal markets share? To avoid raising unfulfilled expectations, I will state at the outset that this paper finds no tidy answer. Illegal markets are not tied together by evolutionary history, and the taxonomic angle is just a metaphor.

Taxonomy not only distinguishes things within a class from those outside the class, it also divides species within the class into orders, families, and genera. In that spirit, I propose below a typology that distinguishes four groups of illegal markets, based primarily on the motivation for prohibiting legal commerce in those goods or services.

## Scope and Definition of Illegal Markets

The scope of inquiry here is the entire supply chain that produces and distributes goods or services whose sale can trigger criminal punishment for at least some market participants. Some adopt a narrower definition, using the term market only when there is a sufficient volume of sales for a typified good that there are well-defined market prices that coordinate supply and demand (2). That may not apply to illegal sales of antiquities, or perhaps even guns (3). We use the term more broadly, as do, for example, economists when referring to a “marriage market” (4).

The qualifier about “some” participants being subject to criminal penalty is necessary because sometimes sellers are punished but buyers are not (e.g., in some drug markets) and sometimes buyers are punished but not sellers (the Nordic model for commercial sex markets). Likewise, we are interested in the entire suite of activities that moves illegally harvested fish to the final consumer, even though such fish are often comingled with legally caught fish in such a way that neither the grocery store nor its customers even know the fish were caught illegally. We pay some attention to the characteristics of consumers, but certainly more could be done along those lines in further work.

As Paoli (5) notes, products do not neatly fall into one of two boxes, either legal or illegal. Legal status varies over time and space. Outside of certain “dry” counties, selling alcohol is legal today in the United States, but it is not now in Saudi Arabia and was not in most of the United States in 1925. Enforcement intensity can also vary sharply. As of this writing, cannabis and heroin are both banned in the United States as “Schedule I substances” but the federal government has a formal policy of noninterference with the operation of state-licensed cannabis companies.

Nor are market participants neatly divided into criminals and law-abiding citizens. Many who derive income from illegal market activities simultaneously work in legal labor markets. E.g., an accountant might both launder money and provide legitimate professional services. The interest here is not only in markets that are “purely illegal,” but rather in all economic activity that supports voluntary transactions for which at least one party may be subject to criminal sanction.

## Comparing Markets and Mammals

There are basically three subclasses of mammals: placentals, marsupials, and the egg-laying monotremes (platypus and echidnas).^*^ Only a few traits are shared by all mammals (hair, milk production, certain skull bones), but if one leaves aside the monotremes, all placentals and marsupials (who are collectively called Therians) share a longer list of traits. That includes live birth, and (with a few exceptions) external ears, protruding noses, and the absence of coracoid bones in the shoulders.

This paper focuses on the illegal market analogs of placentals, which I suggest are markets that operate without cooperation and protection from a state actor, and which sell to large numbers of buyers, whereas the “marsupials” are illegal markets that a government participates in or otherwise supports directly, not just via benign neglect (Table 1).

**Table 1.** Dividing the class of mammals and the class of illegal markets into subclasses

	Mammals	Illegal markets
The familiar subclass	Placentals like cows, lions, whales, and bats	Markets that operate in defiance of government suppression efforts (drugs, money laundering, etc.)
Another major subclass, but different	Marsupials like kangaroos, koalas, wombats, and opossums	Illegal business activity that is supported by state or state-like actors
The odd special cases	Monotremes (egg-laying mammals), namely the platypus and echidnas	A place holder for atypical markets not yet identified

This distinction matters because there are properties shared by all markets discussed in this volume, but not by “marsupial” markets. Those traits are analogous to bone structures that allow the abdomen to expand during pregnancy—something that only placentals need. For example, few organizations operating in defiance of state authority use capital intensive production or distribution processes. Large, valuable assets are too easy to detect and seize if there is a government that wishes to do so.

However, with the protection of a nation state, there can be law-breaking transactions that involve substantial capital equipment. One news article describes the fleet that Russia uses to evade oil sanctions as being a capital asset worth $10 billion (6). In contrast, the typical “lab” synthesizing illegal drugs is a crude basement affair with perhaps a few tens of thousands of dollars’ worth of glassware and reagents. Likewise, illegal arms traffickers selling military equipment can—with the support of nation states—operate with a fundamentally different scale than the common illegal sale of handguns.

Few organizations described in the papers within this volume use expensive capital equipment. The partial exceptions are some factories producing counterfeit goods and illegal, unreported, and unregulated (IUU) fishing operations that employ “reefer” (refrigerated factory) ships to consolidate the catch from distant waters fishing vessels. Notably, those reefer ships operate with the tacit and at times explicit support and protection of nation states, and both factories that make counterfeit goods and fishing boats are dual use—capable of operating legally or illegally—so their existence is not prima facie evidence of criminal conduct.

Most organizations involved in “regular” (placental) illegal markets are small, and the exceptions prove the rule. For example, the United Wa State Army with its 20,000 to 30,000 soldiers has been described as the largest drug-producing organization in Southeast Asia, and its president was identified as a narcotics trafficker under the Kingpin Act (7). However, the United Wa State Army is not just a drug trafficking organization (8). Winn (9) describes it as a narcostate in the same way that Saudi Arabia is a petrostate, i.e., recognizing that it is effectively a state actor.

The focus here is nonstate actors operating in markets that supply goods and services to customers who are either consumers in the literal sense (for drugs, commercial sex, etc.) or illegal businesses that are relatively small (for guns and money laundering services). Illegal markets in which state actors participate directly merit a separate analysis.

### The Striking Variety of Illegal Markets.

Illegal markets manifest striking diversity in size, form, and habits. Some sell physical products (guns, tobacco, drugs, fish, animal products, and counterfeit goods) or digital products (stolen credit card numbers or passwords), whereas others supply services (gambling, in-person commercial sex, money laundering, human smuggling). Money laundering restores to full value money that is damaged (dirty), the same way that repair services fix broken physical objects. Likewise, human smuggling—not to be confused with human trafficking—provides the service of transporting customers across borders that they are not legally allowed to cross.

Some markets sell things for which there is essentially no legal supply (heroin, elephant ivory, money laundering, human smuggling), but in other cases what is illegal is who, how, where, or when the production or distribution is done (IUU fish, counterfeit goods, most wild animal products, loan sharking), and sometimes that varies by jurisdiction (e.g., commercial sex). When both legal and illegal supply coexist, taxes and regulations directed at the legal market can affect outcomes in the illegal market.

Many illegal markets supply commodities (cocaine, pangolin scales), but some offer differentiated goods and services (human smuggling, commercial sex, many antiquities). Many supply chains cross international borders, but not always for illegal guns and essentially never for in-person commercial sex. The customer—and perhaps even the seller—may have crossed a border to reach a jurisdiction with more liberal commercial sex laws, but the production and delivery of the service happen in a single location.

Supply often moves from lower- to higher-income countries, as when animal products harvested in Africa are sold in East Asian markets or methamphetamine is produced in Afghanistan and exported to Australia (10). But there are seizures of methamphetamine bound for Australia from Canada (11), guns produced in the United States that get smuggled into Mexico (12), and counterfeit pharmaceuticals produced in India but sold in Sub-Saharan Africa (13).

Some markets have kingpins who are singularly powerful, but most do not. For that matter, whereas some markets employ “professionals” who derive most of their income from that employment (commercial sex, IUU fishing), in other markets (money laundering, international wildlife trafficking (IWT), guns) selling is commonly a side hustle supplementing other income, legal or illegal.

There are differences of scale. Four or five markets are prominent in many countries and gross $100 billion per year globally (counterfeit goods, drugs, commercial sex, illegal cigarettes, and perhaps gambling). Others are probably in the low tens of billions of dollars (human smuggling, IUU fish, IWT). Social costs can exceed the scale of retail sales. It is hard to attach a dollar value to the cost of gun markets providing criminals with access to firearms or IWT potentially triggering an epidemic of zoonotic diseases.

Some production chains and retail markets exploit large numbers of poor workers (commercial sex, IUU fishing, IWT, and drugs, particularly crop-based drugs), but worker-exploitation is not a central dynamic for money laundering, illegal firearms, cigarettes, or many counterfeit goods or gambling operations. Some illegal markets supply goods or services that are morally taboo (drugs, commercial sex), but others carry little moral stigma (counterfeit goods). Table 2 summarizes some of these differences.

**Table 2.** Dimensions of variation across illegal markets

	Yes/Common	No/Less Common
Product is banned regardless of how it is produced	Heroin, ivory	IUU fish, counterfeit goods, most wild animal products
Transnational supply chains	IWT, IUU fish	In-person commercial sex
Flow is from poor to wealthier countries	Cocaine, IWT from Africa to East Asia	Guns from U.S. to Mexico; counterfeit pharmaceuticals from India to Africa
Kingpins exist	Drugs	Most other illegal markets considered here
Exploit low-skilled workers	Commercial sex, IUU fishing, IWT, crop-based drugs	Money laundering, illegal firearms, cigarettes, or many counterfeit goods
Morally taboo	Drugs, commercial sex	Counterfeit goods

The list could be expanded, but those mentioned serve to make the meta-point. Illegal markets display amazing variety, and salient attributes of one may be absent in another. In the animal kingdom, variation is an evolutionary response to the physical environment and competitive pressures. Within illegal markets, much variation may be driven by the physics of the product and/or by competitive pressures produced by policy and law enforcement.

## Consequences of Physical Attributes of the Illegal Product

Physical attributes of the good or service can drive differences across markets and supply chains. For example, cash to be laundered has a very high value to weight ratio. Paper currency weighs about 1 gram per bill, so when transporting “dirty” cash in the form of $20 bills the value to weight ratio is about $20,000 per kilogram. The ratio is about the same for cocaine and opioids entering final market countries in North America or Europe, and for rhino horns (14). When the denomination is 500 Euro notes, the value to weight ratio is almost 30 times greater.^†^

Few other illegal goods are like that. Even elephant ivory and pangolin scales are more likely to sell in final market countries for $500 to $1,000 per kilogram, not $10,000 to $20,000 (16, 17).^‡^ Firearms and cigarettes are an order of magnitude less valuable per unit weight, closer to $100 per kilogram.^§^ Smuggled humans and IUU fish are even bulkier,^¶^ with prices closer to $10 per kilogram. It is perhaps no coincidence that IUU fishing can involve the most mechanized production among the markets discussed here. The Chinese import price for rosewood—a surprisingly large and important IWT product—is only $1,200/m^3^ or about $1.40 per kilogram (17).^#^

**Fig. 1.**
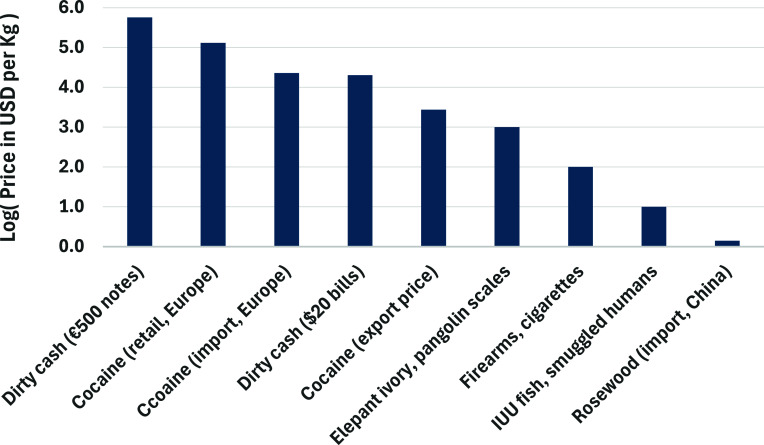
Price per kilogram of different illegal items (USD, log scale).

As Fig. 1 illustrates, the value to weight ratios for illegal products vary enormously. The ratio for 500 Euro notes is about 400,000 times that of Rosewood. That variation helps explain differences in the structure, conduct, and performance of suppliers in those different illegal markets.

High value-to-weight ratios facilitate smuggling because easy-to-conceal quantities can be economically meaningful. At $20,000 per kilo, a courier can carry $200,000 worth of product in a backpack, or $2 million worth in a car trunk. No one smuggles rosewood via couriers on commercial airlines; the fee per checked bag would exceed the value of the smuggled commodity.

Products also differ in terms of perishability. Cocaine purity degrades only moderately over periods as long as 5 y (18), but fresh khat leaves are highly perishable, so trafficking is regional, mostly confined to the Horn of Africa and Arabian peninsula, near where it grows (19). It is perhaps no coincidence that many internationally traded illegal goods are shelf-stable. Live animals (e.g., for exotic pets) are an exception, as is fresh fish.

Other differences in “physics,” broadly construed, relate to consumers. Most consumers of human smuggling services hope to purchase only one journey in their lifetime.^||^ At the other extreme, people with opioid use disorder may purchase illegal fentanyl multiple times per day, and so more than one thousand times per year. On that spectrum, people making illegal purchases of guns, antiquities, and counterfeit goods are closer to human smuggling customers, making purchases only occasionally, whereas big spenders on (both legal and illegal) gambling are more like heavy drug users in terms of purchasing frequently.

Those realities can shape the architecture of distribution networks. Drug distribution networks have to support very high-frequency purchasing. That limits the time that can be spent hiding any given retail transaction, and so exposes retailers to police enforcement. That in turn creates incentives for having multiple layers of intermediaries separating kingpins from retail sellers. And that keeps retail prices high because every transaction layer adds cost. By contrast, purchases of antiquities are infrequent, high-value, even bespoke, so the transactions can be discreet.

Another distinction pertains to the share of consumers’ disposable income that gets spent on the product. Most “hard drugs” are purchased by people who spend a large share of their income—often half or more—on drugs. Compulsive gamblers and some customers of human smugglers may be the only other consumers of illegal products who come close. Money laundering customers, blue collar pack-a-day smokers, and people in Ghana who depend on IUU fish as a food staple may spend 5 to 15% of their income on those products, but a dealer who makes $50,000 selling drugs while buying one $500 illegal handgun per year spends only 1% of income on illegal gun purchases.

Together those “physical” factors help explain why markets for expensive drugs are so distinctive. They are perhaps the only markets whose modal customer spends the majority of their disposable income making daily purchases of products with a very high value-to-weight ratio.

The number of retail transactions is far lower for durable goods (e.g., guns, ivory, antiquities), so those retail markets are rarely a nuisance to neighbors. The marketplaces for illegal services sometimes generate local externalities, particularly “street” markets for commercial sex but also the congregation of customers seeking human smuggling services.

## Policy and Enforcement as Drivers of Variation

Production and distribution adapt and respond to enforcement pressure. Indeed, the dynamic interaction between smugglers and law enforcement has been described as an “arms race” vis a vis human smuggling (20), drugs (e.g., ref. 21), and technology-enabled smuggling more generally (22). Adaptation at the retail level was suggested by the responses to two questions asked of all RAND PRIM workshop attendees: “Are consumers mostly ignored by law enforcement?” and “Are retail transactions open (flagrant)?” The answers matched in every market except illegal wildlife, for which the match was partial (yes consumers are mostly ignored, but only “sometimes” are retail sales flagrant). When consumers are subject to low enforcement risk, retail markets can be flagrant; when it is policy to arrest consumers, the retail markets become covert.

Reuter and Kleiman’s (23) “Risks and Prices” model is one of the most important theories concerning illegal markets. It posits that price markups from one market level to the next are so high to compensate suppliers for their risks of enforcement and violence. The theory was developed for U.S. drug markets, and workshop participants believed it explained the high prices charged by money launderers and human smugglers, but that it did not apply as much to suppliers of commercial sex, IUU fish, or IWT. That lack of applicability actually supports the Risks and Prices theory, since in those three markets the suppliers’ risks of incarceration are lower.

Most market experts reported that governments invest nontrivial resources in seizures and market disruption. The partial exceptions include counterfeit goods, IWT, and commercial sex in some places. (And of course in places where the market is legal—cannabis in Canada, commercial sex in Germany—enforcement is limited to suppliers who are unlicensed or otherwise operating outside of the legal regime).

Most market-experts reported that there were what Reuter (24) describes as “structural consequences of product illegality”—meaning that the mere fact of prohibition plus at least a modicum of enforcement forces suppliers to operate in ways they would not, but for that illegality. E.g., sneaking across an international border is fundamentally different than getting one’s passport stamped while flying into a major airport, and it remains so during times of greater and lesser investment in border patrols.

In short, illegality and enforcement can force adaptation,^**^ and often that adaptation drives up prices. As Kilmer notes (25), parcel delivery services will transport a (legal) 1-kg box from Colombia to London for less than $150, but the convoluted routes and methods employed by drug traffickers create an $18,000 per kilogram markup between Colombia and Europe (25).

Commercial sex may be a partial exception. Structural consequences of illegality affect the nature, location, and particulars of commercial sex, but the illegal service is not necessarily more expensive. Indeed, it can be less expensive.

It is not clear why. One speculation is that legal regulation may benefit the weaker party in a transaction. In many illegal markets, the seller has the upper hand. Relative to that situation, a regulated legal market may favor buyers, including with lower prices. However, in commercial sex markets, often the seller/supplier is the vulnerable party. Perhaps in that case, a regulated legal market favors the seller, including via higher prices. That is just a speculation, and needs supporting examples, but it is consistent with workers benefitting from minimum wage laws prohibiting the sale of labor services at low prices.

## The Pace of Innovation

The fact that supply chains have adapted says nothing about the speed—or lack thereof—with which they adapt. Horses are well adapted to life on the prairie, but that took multiple millions of years. Every expert at the conference rated “their” market as adapting at least fairly quickly in two respects: 1) Adapting to changing enforcement tactics and 2) Taking advantage of new, modern technologies and opportunities. Examples of the latter include rapid adoption of burner phones, encrypted communication, cryptocurrencies, dark web sales portals, and delivery drones. One can speculate that this may be a result of the fragmented, competitive nature of most illegal marketplaces, as well as survival bias—with those who do not adapt getting arrested.

By contrast, there was considerable variation in the extent and pace of innovation in product forms and core production methods. Drug markets illustrate this. Since 1970 there have been only a limited number of major product innovations in illegal drug markets,^††^ but after legalization, there was an explosion in cannabis product forms (vapes, dabs, drinks, infused prerolls, lotions, etc., as well as Delta-8 and balanced CBD-THC products). Similarly, there has been an explosion in sports gambling product offerings in the United States after its legalization via the 2018 Murphy vs. NCAA Supreme Court decision.

Apart from ghost guns (firearms that are privately assembled, often from kits), there has been relatively little product innovation in illegal firearms markets, and IUU fishing methods have evolved mostly by adopting new methods from the legal fishing sector, not by inventing them specifically for IUU fishing. So those illegal markets could be rated as being low to fairly low on internally generated invention of new products or core process technologies.

In contrast, the “world’s oldest profession” has seen enormous innovation in recent years, with a considerable share of commercial sex work moving online via sites such as OnlyFans. That new form is illegal in some places, legal in others, and inhabits a gray area in still others. Regardless of whether one views those sites as an innovation within an illegal market, or the creation of a legal substitute, it is a fundamentally new product form, not just the legalization of something that had already existed. On-line commercial sex platforms also change the relationship between sellers and customers (e.g., now including one-to-many service delivery and subscription-based payment), and the geography of selling (international and other long-distance service delivery becomes possible).

Innovation in the human smuggling sector has also been fundamental, and now offers protections for customers that may help explain growing sales volumes. For example, it is now much easier to arrange payment upon successful delivery of services rather than payment in advance, and also to arrange payment by third parties. Both reduce victimization of customers who might otherwise have to carry large amounts of cash. Cheap, secure international communication also offers greater scope for preplanned and bespoke human smuggling services. One could argue that this is just adopting modern banking and communication technologies, but it is doing so in a way that changes fundamental dynamics of price, payment, and delivery of services. Money laundering can also look quite different today, including because of cryptocurrencies, although old fashioned methods persist.

Some changes in IWT are technological (e.g., using drones in poaching), though many pertain to broader changes in macroeconomic, demographic, and foreign policy trends. For example, the explosion in the number of affluent people in East Asia, and China’s heavy foreign direct investment in Africa, have greatly expanded smuggling opportunities for African animal products being used in Traditional Chinese Medicine (TCM).

Overall, when the experts compared markets in 2025 to those 20 y earlier, it was easy to see both considerable continuity and considerable change. Many illegal markets seem broadly similar, but bigger and/or more challenging than before,^‡‡^ but the illegal markets for e-cigarettes in Australia and some other countries are entirely new. Such devices simply did not exist back in 2005.

## Traits Shared by All, or Almost All, of the Nine Markets

Having discussed prominent features that are *not* shared by all illegal markets, and forces that spawn that variety, the next question is what are common features? The experts agreed that the markets tend to be “demand driven.” Suppliers invest little in marketing to drum up demand, and sellers rarely push random strangers to buy something that they do not already want.

Fentanyl may be atypical in this regard. The illegal markets did not promote its demand, but the transition from heroin to illegally manufactured fentanyl in North America appears to have been motivated by suppliers’ interests, not by consumer demand.

Demand can, though, surge, as with the epidemic spread of cocaine in the late 1970s in the United States (26) or when war in Syria induced millions of refugees to pay human smugglers to take them to a safer place. UNODC (17) describes an interesting example of a new orchid being discovered in Vietnam in 2009. After pictures were posted on social media, demand surged, driving prices up to $100 per plant, but then prices crashed, down to $10 per kilogram, as the craze passed. Likewise, demand for pangolins from one country can surge when the animal’s population in another country gets exterminated and suppliers shift to a new source.

Monopoly power is rare, and any exceptions tend to be local. Extending credit is also rare, perhaps because lenders cannot rely on courts to help collect from delinquent creditors. Sometimes human smugglers and drug dealers extend credit to customers, but even in those markets, it is not the norm. Perhaps drugs’ markets differ because customers are often cash-poor and high-frequency transactions help solve the trust problem (27, 28).

Regarding responses to enforcement, in all or almost all markets: 1) Law enforcement tries to seize products and/or disrupt markets, 2) This alters the way production and supply chains operate, 3) Supply chains adapt quickly to changing enforcement tactics, and 4) Organizations and markets can expand quickly if demand increases, or if other suppliers are eliminated. The last point is crucial for policy and is discussed further by Paoli and Reuter (1).

There are other commonalities that were not discussed directly at the RAND PRIM workshop. Few suppliers of illegal products invest much in research and development, let alone the patenting of innovations. Drug traffickers will hire “chemists,” but they are often only trained at the undergraduate level, and “discover” new drugs and synthesis methods more by searching the internet than by inventing something entirely new.

There is little branding. Even in counterfeit goods markets, the brand (e.g., Gucci for a handbag) is not the brand of the actor supplying what is illegal. Sometimes drugs are sold with a stamp, label, or name that supposedly marks a particular good batch (29, 30). However, those signals are often ephemeral and mistrusted, perhaps because they are easy for others to copy. That shows a desire to differentiate one’s product, but also the limited success drug sellers have at establishing an enduring brand.

Many illegal markets are not supported by multilayered distribution chains; commercial sex, for example, is not. However, when many transactions do separate producers from final consumers, there are usually very large increases in price as one moves down the distribution chain. Kilmer (25) documents this for counterfeit opioid pills and cocaine, noting that retail prices can be a hundred times the production cost. Likewise, the Pangolin Reports (31)—though not a refereed academic source—describes pangolin scale prices of $5 to $15 per kilogram in Africa (which is less than the price per kilogram of pangolin meat), but rising to $450 to $600 per kilogram in Viet Nam, Hong Kong, and China.

## Illegal Market Suppliers Do Not Resemble Corporations

Many of these common traits can be distilled into a more concise statement. Illegal market suppliers generally do not look or behave like corporations. E.g., a recurrent theme is the relative absence of corporate organizational forms and behaviors such as branding, marketing, and research and development. The staff of illegal enterprises are also not generally salaried full-time workers. Except for crews on IUU fishing boats, many are more like gig workers, paid by the task.

This is similar to the traditional observation that illegal markets are usually supplied by networks of small and informal enterprises. Certainly, that characterization is preferred to the myth that illegal supply is dominated by monopolists and cartels; even the infamous Medellin drug cartel was unsuccessful at preventing cocaine prices from collapsing during the 1980s (32). However, not all illegal suppliers are small. Some IUU fishing enterprises employ refrigerated cargo vessels to transport illegally caught fish to distant ports with pliant inspectors. And the network image fits better with IUU fishing, IWT, and drug distribution than it does with commercial sex. There are networks for human trafficking, but as Shah (33) explains, that is a different activity.

The ownership structure of criminal enterprises is usually a sole proprietorship or partnership. Profits are not shared by a diffuse set of shareholders who hire professional managers to run the operation. Again, some forms of IUU fishing are a possible exception because it can be companies that do long-distance illegal fishing.

Other exceptions include street gangs, mafias, and outlaw motorcycle groups that serve functions above and beyond producing and distributing illegal products. That can include governance-type criminal organizations and those that serve some social purpose (protection for members, sense of belonging, etc.) (34, 35).

One hypothesis is that governance-type organizations outcompete more specialized trade-focused organizations when the governance-type organizations have other reasons to exist (36). That may enable them to directly challenge smaller trade-organizations or to achieve efficiency by adding the illegal market activity to a suite of other activities that collectively “amortize” the “overhead” of, for example, corrupting or coopting enforcement agencies. But much data would be needed to test that hypothesis.

The scarcity of corporations in illegal markets may translate into a deficit of those activities that corporations do well. That includes activities that require large upfront outlays in order to reap a stream of future income, such as investment in capital-intensive facilities, R&D projects, and development of brands.

Note, even if the absence of corporations alters the structure, conduct, and performance of illegal markets, it does not eliminate supply, because corporations are not the only option. There are still family-owned restaurants; some groceries are bought at farmers’ markets; and for every Cirque du Soleil or Disney theme parks division, there are myriad live performers who are effectively individual proprietors.

One reason why the enterprises supplying illegal products do not look like corporations is their inability to enforce contracts in courts of law (24). The underlying legal principle is “*Ex turpi causa non oritur action*” or “No court will lend its aid to a man who founds his cause of action upon an immoral or an illegal act.” This principle, which dates to the 1775 English court case of Holman vs. Johnson, not only affects interactions between the retail seller and customer, e.g., making sellers reluctant to extend credit. It can ripple up the supply chain affecting interactions between parties that the customer never sees.

The inability to enforce contracts may favor repeated simple, relatively low-value transactional interactions relative to long-term relationships that require trust (37). Elementary trades can happen even without recourse to courts or arbitration, particularly when the benefits of repeated future interactions outweigh what could be gained by defrauding the other party in any single transaction. Such trading has happened throughout history, even when the trader operates far from home (e.g., 17th century fur traders operating far from their bases in Montreal or the shores of Hudson’s Bay).

Complicated relationships—such as sharing ownership across many people or getting teams to coordinate on activities when individual contributions are hard to measure—are harder to institutionalize without a stable legal structure for resolving disputes.

Hence, while illegality may favor small organizations over vertical or horizontal integration, that may be a symptom of a more fundamental issue. Inasmuch as illegality favors simple transactional interactions, it undermines the benefit to suppliers of adopting a corporate structure.

Some modern technologies that illegal markets have been quick to adopt can be seen as extending the domain of simple transactional interactions. For example, dark web marketplaces (e.g., Silk Road and its successors) reduce to a simple transaction a trade that can span thousands of miles, and cultural and linguistic differences between parties to the transaction. The dark web marketplaces’ escrow services and posting of customers’ reviews of suppliers (and suppliers’ reviews of customers) help overcome barriers to trust. They also permit a limited degree of advertising without creating vulnerability to enforcement.

## A Typology of (Most) Illegal Markets

Taxonomy both distinguishes things within a class from those outside the class, and divides species within the class into orders, families, and genera. Here I propose that most “placental” species of illegal markets (meaning those that operate without explicit protection of, or participation by, a government) can be grouped into four orders.

### Production by Theft.

Many illegal markets are supplied via production that is essentially stealing from a common heritage, either natural or cultural. Examples include IUU fishing (38), IWT (39), and illegal mining of resources both physical (gold, sand, etc.) and cultural (antiquities). The core problem is that production is not sustainable, either because the resource is finite (antiquities) or harvesting exceeds the natural regeneration rate (e.g., poaching of slow to reproduce elephants).

Often illegal harvesting exists alongside regulated, legal harvesting, such as sustainable fishing within legal quota limits or recovery of antiquities by professional archaeologists working for national museums. So often downstream in the supply chain there can be challenges determining whether a particular item was produced illegally or not. For example, Poots (40) describes how New York City’s Metropolitan Museum of Art unwittingly paid $4 million for a golden coffin from Pharaonic Egypt that had been dug up by grave robbers in 2011.

Generally, production will locate where the state is either ineffectual or uninterested in protecting that resource, and international distribution moves from the global south to middle- and high-income countries. That can raise complicated ethical questions of who has the responsibility and the right to police production and trafficking.

### Supplying Goods and Services That Are Used to Commit Other Crimes.

A second group of illegal markets sells products that are banned because their principal use case is aiding or abetting the commission of crimes beyond just consuming banned products. Money laundering is the paradigmatic example (41). Mostly the people who pay to launder money are people who acquired money illegally.^§§^

Some gun prohibitions belong in this category (42). E.g., one reason for banning convicted felons from possessing firearms is to deny access to someone who might threaten or commit violent acts with that gun. Likewise, laws prohibiting the sale of stolen credit card numbers and fake identities hope to deny criminals access to those crime-facilitating tools.

One can broaden the category to include products that help people violate other laws, not just criminal codes. For example, there are markets for fake ID cards that allow underage individuals access to places where alcohol is served. Andric and Kilgannon (43) describe overseas websites selling high-quality fakes, and a supplier to such a site making a single sale of $1.3 million for 30,000 fake IDs. Likewise, Virginia’s ban on the use of radar detectors extends also to their sale within the Commonwealth.^¶¶^

### Markets That Undermine a Legitimate Authority’s Rights.

A third group is market activity that undermines the rights of a specific third party, either a private rights holder or the government. Many transactions involving counterfeit goods fall in this category (44). When counterfeiters sell a knock-off handbag with a Gucci label, Gucci loses the fruits of its intellectual property. Likewise, human smugglers undermine nation states’ authority over their borders (45), and selling untaxed cigarettes undermines the authority of government to collect taxes (46).

### Goods and Services That Harm the User and/or Seller.

The final category is goods and services whose consumption directly causes harm or, less commonly, whose production harms workers. Addictive substances are the paradigmatic example (25). Their prohibitions can have many motivations (religious taboos, racism, etc.), but consumption-related harms to consumers and third parties are often central. The precise profile of risks can vary. Opioids carry a particularly high risk of overdose. For powerful stimulants like cocaine, crack, and methamphetamine compulsive use and intoxication-induced paranoia and violence may loom relatively greater. Tobacco bans (e.g., against e-cigarettes in Australia) aim to protect consumers and people exposed second-hand from heart disease, lung cancer, emphysema, and other harms from chronic exposure.

Prohibitions against gambling also seek to prevent self-harm, though the proximate harm may be more damage to family finances than direct health harm. Bans on some counterfeit products, including medicines, not only protect the rightful patent-holders’ profits, but also the safety of people who might be harmed by substandard—or completely phony—medicines (44).

Prohibitions against commercial sex have multiple motivations including moral considerations, and perhaps commercial sex belongs in its own order (33). However, one consideration is concern about harms the seller might experience, even if the trade were legal.

This last category of prohibitions presents peculiar policy challenges because a primary goal is protecting one of the willing participants in the transaction from their own choices. None of the other three categories are victimless. The victim may be diffuse (cultural heritage, government authority, or the environment) or not party to the transaction (as with counterfeit goods), but those crimes go unreported because there is weak guardianship, not because there is no victim.

The special character of this last order creates three complications. First, the proximate effect of enforcement is to harm one of the parties of the transaction, but the purpose of the prohibition is to help those individuals. That is an inherent tension.

Second, for the state to enforce this type of prohibition, it must inject itself between two willing parties, not just protect an external interest. That makes enforcement intrinsically intrusive.

Third, these prohibitions tend to have a greater moral dimension than, say, prohibitions of counterfeit goods or money laundering. The motivation for the prohibition is a collective judgment that the activity is harmful to the parties themselves. Absent that judgment, there would be no cause for prohibition. Given that judgment, the continued participation in the market seems unnatural and easy to condemn.

### Observations About Other Illegal Markets.

The major orders of placentals (carnivores, rodents, bats, primates, even and odd toed ungulates, whales) omit important special cases (elephants are in their own order) and some that get little thought. (Hedgehogs, hyrax, and aardvarks each get their own orders too.)

Not all illegal markets fall neatly into the four categories just described. Many countries make it illegal to sell surrogate pregnancy services, but others (including the United States) do not. That leads to the paid supply of this banned service and so a market, albeit a small one. It is not clear whether bans on paid surrogate pregnancy are intended to protect the seller (as with bans on commercial sex), to protect the aggregate cultural good (avoiding the commercialization of something that “shouldn’t” be traded in a capitalistic market), to protect the child, or someone else.

What is more interesting than chasing down the tree shrews of the world (yes, they too get their own order) is considering instances in which similar looking beasts end up in different orders for good and fundamental reasons.

Many prohibitions pertain to food products. In all or parts of the United States, horse meat, foie gras, unpasteurized (“raw”) milk, haggis, sea turtles, and fugu fish are all essentially banned. However, not all of the resulting ban-evading illegal supplies belong in the same order. The prohibitions on fugu fish and raw milk—like the prohibition on heroin—are primarily intended to prevent consumers from harming themselves via their own choices. That is not to say that all fugu fish and raw milk consumers suffer harm, or even that the bans are harm-reducing. Rather, it is just a statement about the intentions or motivations of the ban.

By contrast, the U.S. federal ban on eating sea turtle meat or eggs is like the ban on buying elephant ivory; it is intended to protect the environment.

Bans on horse meat and foie gras belong in yet another category, perhaps one related to maintaining purity (47), since neither are dangerous for the consumer or the environment. (The U.S. horse meat ban pertains to domesticated not just wild horses.)

Thus, despite being superficially similar, bans on various food products belong in different orders, just as voles, moles, and golden moles all belong in different orders (Rodentia, Soricomorpha, and Afrosoricida, respectively) even though they all look like little furry nuisances to the average person.

Also, just as biological orders get divided into families and genera (there are 15 families of carnivores), the four orders above can be further subdivided. Perhaps intoxicants are a family within the 4th order just discussed, and they can be further divided into separate genera for crop-based vs. synthetic drugs.

## Summary

The discussion above can be summarized in the following 10 conjectures.

1. Illegal markets display enormous variety in the structure and conduct of the systems of enterprises that supply them.
2. Two drivers of that variety are the physics of the good or service being provided and the need to respond to enforcement pressure.
3. Many features that are distinctive for one or several illegal markets are not shared by all. A student of drug markets might think that violence plays an important role in most illegal markets, but that is not the case.
4. The markets described in this special issue belong to a subclass in which supply chains operate in defiance of state control. There are also illegal markets in which state actors actively participate.
5. Many illegal markets have changed fundamentally over the last 20 y. Since 2005, the illegal market for cannabis in Canada has largely been replaced by legal supply, but the invention of e-cigarettes has created new illegal markets where they are banned (in Australia, for example). Online markets for sex work are also entirely new. Their legal status is ambiguous and evolving, but they compete with and affect the markets for traditional illegal sex work. Many other salient changes stem from illegal markets adopting technological innovations that were invented by and for the legal economy, rather than by inventing new products or production methods within the illegal economy.
6. The enterprises supplying illegal goods and services do not resemble the corporations that supply many legal consumer goods and services. They generally do not advertise, promote brands, or conduct R&D. A greater share of staff are gig or piece rate workers, not salaried full-time employees.
7. This stems in part from the legal principle of ex turpi causa non oritur actio, which makes contracts made in the furtherance of a criminal enterprise unenforceable in courts.
8. The nature of the harm motivating bans that create the different illegal markets can vary. It is production not consumption of elephant ivory and rhino horns that create harms, whereas it is consumption not production of fentanyl and gambling that can create harm.
9. One grouping of the common illegal markets distinguishes: i) Supply of goods stolen from a common heritage, either natural (IUU fishing, IWT, illegal mining of gold or sand) or cultural (the antiquities trade). ii) Aiding and abetting other crimes by supplying goods and services to criminals (guns, money laundering). iii) Crimes that undermine a legitimate authority’s rights (smuggling humans across borders, counterfeiting goods, tax-evading smuggled cigarettes), and iv) Goods and services that harm the user or seller (drugs, commercial sex, gambling, raw milk, Australia’s ban on e-cigarettes).
10. Only the fourth type of illegal market can potentially be considered victimless crimes. They create particular policy challenges because enforcement presumes a paternalistic logic, but nonetheless harms individuals the prohibition seeks to help. It is also inherently intrusive because it injects the government between two willing parties whose actions are not directly harming other, third parties.

These hypotheses are tentative first steps. Hopefully they will be tested, extended, corrected, and supplanted by further inquiry, including by paying greater attention to the customers or demand side of these markets and by tapping other metaphors. For example, just as convergent evolution can produce similar physical traits in taxonomically distant species, say a mammal and a reptile that fill the same ecological niche, perhaps similar policies or enforcement pressures can create commonalities in the markets for very different products, say one a good and the other a service. It might also be interesting to catalog all of the ways in which these markets do or do not differ from what might be predicted by a range of standard economic models. The more they differ, the greater the potential need for new models; the less they differ, the more we might rely on standard models, albeit with grounding in contextual factors specific to the given market. Still, however open to improvement, these ten ideas do still illustrate some potential benefits of thinking about illegal markets as a class, not just studying individual markets in isolation.

Just as one cannot understand a river by examining it a bucket at a time, or understand the mammalian class when studying only pachyderms, one cannot truly understand illegal markets through mastery of just one.

## Data Availability

There are no data underlying this work.

## Footnotes

^*^Comparisons made here between classification of markets and mammals are done at an elementary level. That harnesses a metaphor that can resonate with readers with diverse backgrounds, but may at times pain professional taxonomists. It might be more accurate to divide mammals into two *subclasses*, the monotremes and Theria, with the division of Theria into placentals and marsupials pertaining to infraclasses, not subclasses, but that degree of precision is not helpful for present purposes.

^†^Soudijn and Reuter (15) study couriers who carried as much as 400,000 Euros in 500-Euro notes.

^‡^Cannabis prices vary considerably by form (resin vs. herb), location, and legal status, as well as over time, but are often somewhere between $1,000 and $10,000 per kilogram, and so are intermediate between the expensive illegal drugs (cocaine, heroin, etc.) and other commodities.

^§^There is great variation in these markets, but: If a 7-pound assault rifle sells for $1,000 that is $65 per kilogram. If a 20-g pack of cigarettes wholesales for $4, that is $200 per kilogram.

^¶^If smugglers charge $5,000 to transport a 150-pound human that is $15 per kilogram. Wholesale fish prices are often less than $10 per kilogram. Even bluefin tuna is often only $40 to $80 per kilogram, although when one particularly prized bluefin was auctioned for $3.1 million in 2019, that worked out to more than $2,000 per kilogram.

^#^Counterfeit goods vary on this spectrum. If a 0.4 kg knockoff Gucci handbag ever sold for the same price as its genuine twin, that could be $4,000/0.4 kg = $10,000 per kilogram—close to the price of drugs and rhino horn. But if it sold on a sidewalk for $40, that would be only $100 per kilogram.

^||^The journey may involve multiple border crossings, but they hope to cross each border only once.

^**^Tax-evading cigarettes may be a partial exception. They are identical to legal cigarettes, and distribution methods are not all that different; movement is by truck and sale often at or just outside of convenience stores, at least in the U.S.

^††^These include crack supplementing powder cocaine, sinsemilla displacing “commercial grade” cannabis, fentanyl displacing heroin from North American opioid markets, and methamphetamine joining cocaine/crack, cannabis, and heroin as one of the “big four.” There are also now myriad “new psychoactive substances” (NPS), but they collectively account for a quite minor share of sales volume.

^‡‡^IUU may be an exception. Its sales may be past peak, but that is in part because fish stocks are getting depleted, not only because control policy has become more effective.

^§§^In countries with oppressive and incompetent regimes, sometimes otherwise law-abiding individuals pay to convert currencies, but even in that case the consumers of money laundering services can be criminals in the government’s eyes.

^¶¶^§ 46.2-1079. Radar detectors; demerit points not to be awarded.
